# Basal Forebrain Cholinergic Activity Modulates Isoflurane and Propofol Anesthesia

**DOI:** 10.3389/fnins.2020.559077

**Published:** 2020-10-27

**Authors:** Tian-Yuan Luo, Shuang Cai, Zai-Xun Qin, Shao-Cheng Yang, Yue Shu, Cheng-Xi Liu, Yu Zhang, Lin Zhang, Liang Zhou, Tian Yu, Shou-Yang Yu

**Affiliations:** ^1^Department of Anesthesiology, Affiliated Hospital of Zunyi Medical University, Zunyi, China; ^2^Guizhou Key Laboratory of Anesthesia and Organ Protection, Zunyi, China; ^3^Key Laboratory of Brain Science, Zunyi Medical University, Zunyi, China

**Keywords:** propofol, isoflurane, basal forebrain, cholinergic neurons, anesthesia, consciousness

## Abstract

Cholinergic neurons in the basal forebrain (BF) have long been considered to be the key neurons in the regulation of cortical and behavioral arousal, and cholinergic activation in the downstream region of the BF can arouse anesthetized rats. However, whether the activation of BF cholinergic neurons can induce behavior and electroencephalogram (EEG) recovery from anesthesia is unclear. In this study, based on a transgenic mouse line expressing ChAT-IRES-Cre, we applied a fiber photometry system combined with GCaMPs expression in the BF and found that both isoflurane and propofol inhibit the activity of BF cholinergic neurons, which is closely related to the consciousness transition. We further revealed that genetic lesion of BF cholinergic neurons was associated with a markedly increased potency of anesthetics, while designer receptor exclusively activated by designer drugs (DREADD)-activated BF cholinergic neurons was responsible for slower induction and faster recovery of anesthesia. We also documented a significant increase in δ power bands (1–4 Hz) and a decrease in β (12–25 Hz) power bands in BF cholinergic lesioned mice, while there was a clearly noticeable decline in EEG δ power of activated BF cholinergic neurons. Moreover, sensitivity to anesthetics was reduced after optical stimulation of BF cholinergic cells, yet it failed to restore wake-like behavior in constantly anesthetized mice. Our results indicate a functional role of BF cholinergic neurons in the regulation of general anesthesia. Inhibition of BF cholinergic neurons mediates the formation of unconsciousness induced by general anesthetics, and their activation promotes recovery from the anesthesia state.

## Introduction

General anesthetics, including intravenous and inhalation ones, have been widely used since the 1840s, yet there have been no common mechanisms explaining how general anesthetics induce the sudden reversible loss of consciousness. As the sedation effects of anesthetics such as calmness, drowsiness, and muscle relaxation are behaviorally similar to the features of sleep especially in the non-rapid eye movement (NREM) period (Murphy et al., 2011; Li et al., 2018), general anesthesia has been described as like sleep (Franks and Zecharia, 2011). Moreover, some whole-brain imaging studies indicated that the state of “unconsciousness” during deep sleep and general anesthesia is remarkably similar (Franks, 2008). Thus, the study on the sleep–wake pathway may help elucidate the mechanism of general anesthesia.

The basal forebrain (BF), a large heterogeneous structure in the ventral forebrain, contains cholinergic and non-cholinergic cell populations. It receives ascending projections from the ascending reticular activating system (ARAS) and in turn projects to the cerebral cortex (Brown et al., 2012; Yang et al., 2017). It has been proved to be a crucial site in fundamental support of wakefulness and cortical activation (Anaclet et al., 2015; Xu et al., 2015). In particular, cholinergic neurons in the BF have long been considered to be the key neurons in the regulation of cortical and behavioral arousal (Poulet and Crochet, 2018). Stimulation of the BF area increases cortical acetylcholine and promotes high-frequency cortical electroencephalogram (EEG) oscillations (Han et al., 2014). Prefrontal cholinergic stimulation, rather than noradrenergic activation, induces wake-like behavior in anesthetized rats (Pal et al., 2018), while the prefrontal cortex receives BF cholinergic neuronal projection (Do et al., 2016), suggesting that the BF cholinergic system may play a certain role in general anesthesia. The lesions of BF cholinergic neurons in rats have been shown in previous studies to enhance the effects of anesthesia (Leung et al., 2011, 2013), but the effect of anesthetics on the activity of BF cholinergic neurons has been neglected. Also, the lesion method may lead to changes in sleep debt, thus it will inevitably affect the evaluation of anesthetic efficacy (Moore et al., 2012). With the development of fiber photometry, designer receptor exclusively activated by designer drugs (DREADD), optogenetics, and other methods in recent years, it is possible for us to reveal the real-time effect of general anesthetics on BF cholinergic neurons *in vivo* and further figure out whether their activation could directly arouse anesthetized mice.

In this study, the role of BF cholinergic neurons in isoflurane and propofol anesthesia was evaluated. ChAT-cre transgenic mice were employed to express cre recombinase exclusively in cholinergic neurons. Through real-time fiber photometry system combined with a genetically encoded Ca^2+^ indicator, we found that the activity of BF cholinergic neurons is closely related to anesthesia state. To further explore how neuronal activation of BF cholinergic neurons mediates general anesthesia, we employed three approaches to manipulate BF cholinergic neuron activity. The genetic lesion approach impairs neuronal activity for the long-term, the DREADD approach boosts the activity for the short-term, and optogenetics creates instantaneous neuronal activation.

## Materials and Methods

### Animals

The animal protection and utilization committees of Zunyi Medical University, Guizhou, China, approved all the experimental procedures for this study, and the study followed the guidelines for the care and use of experimental animals in China (No. 14924, 2001). Adult (8–12 weeks, 20–25 g) ChAT-IRES-Cre male mice were used for all the experiments. Under the control of the ChAT gene promoter, Cre recombinase was expressed in ChAT-IRES-Cre mice. All animals were fed adaptively in conditions with an ambient temperature of 22°C ± 0.5°C and relative humidity of 60% ± 2% and were given automatically controlled light, following the principle of 12 h light/12 h dark cycle (lights on at 8:00 a.m.). Food and water were supplied *ad libitum*.

### Stereotactic Surgery Procedure

Mice were anesthetized with pentobarbital [1%, 50 mg/kg, intraperitoneally (i.p.)] before placing them on a stereotaxic instrument (RWD Life Science, China). During the surgery and subsequent tests, a heating pad with a rectal temperature probe was used to keep the mouse body temperature at 37°C. Lidocaine (1%, 0.1 ml) was hypodermically infused on the surface of the exposed skull for local anesthesia. For calcium fiber photometry (*n* = 10), AAV virus (AAV-Ef1α-DIO-GCaMPs) was injected (400 nl, 40 nl/min) unilaterally into the BF [anteroposterior (AP): +0.1 mm, mediolateral (ML): ± 1.3 mm, dorsoventral (DV): −5.4 mm, as per the mouse atlas of Paxinos and Franklin, 2001] *via* a fine glass pipette (1-mm glass stock, tapering to a 10–20 μm tip) through a microsyringe pump (Legato 130, KD Scientific, United States). The pipette was maintained in the BF for 10 min to allow the virus to diffuse, and then slow revocation. After the injection was completed, the optical fibers (200 μm OD, 0.37 numerical aperture, Newton Inc., Hangzhou, China) were put 100 μm over the injection site and fixed on the skull with a transcranial screw and dental acrylic.

For the cholinergic neurons genetic lesion experiment, the virus (AAV-CAG-DIO-DTA) or saline (*n* = 9 for each group) was injected into the BF bilaterally, and EEG electrodes were placed into the skull (AP: +0.1 mm, ML: ± 1.5 mm; AP: −3.5 mm, ML: ± 1 mm) simultaneously (Pick et al., 2011; Kortelainen et al., 2012). All the electrodes were connected to the same microconnector and cemented to the skull with dental cement. The mice were given 2 weeks to recover before subsequent experiments. For DREADD experiments, we implanted EEG electrodes in AAV-Ef1α-DIO-hM3Dq-mcherry and AAV-Ef1α-DIO-mCherry virus (*n* = 8 for each group)-injected mice. The AAV-Ef1α-DIO-hChR2-EYFP or AAV-Ef1α-DIO-EYFP virus (*n* = 7 for each group) were infused bilaterally, and the optical fibers (200 μm OD, 0.37 numerical aperture, Newton Inc., Hangzhou, China) were implanted at 100 μm over the injection site and fixed them on the skull with a transcranial screw and dental acrylic in optogenetic experiment. To avoid confounding effects due to clock shifting (Orts-Sebastian et al., 2019), we performed follow-up experiments between 14:00 and 17:00 after the mice were given at least 3 weeks to recover. At the end of all the experiments, the virus injection location was verified by immunofluorescence staining combined with mouse brain atlas (Paxinos and Franklin, 2001), and data with incorrect modeling positions were eliminated.

### Calcium Fiber Photometry

**FIGURE 1:**
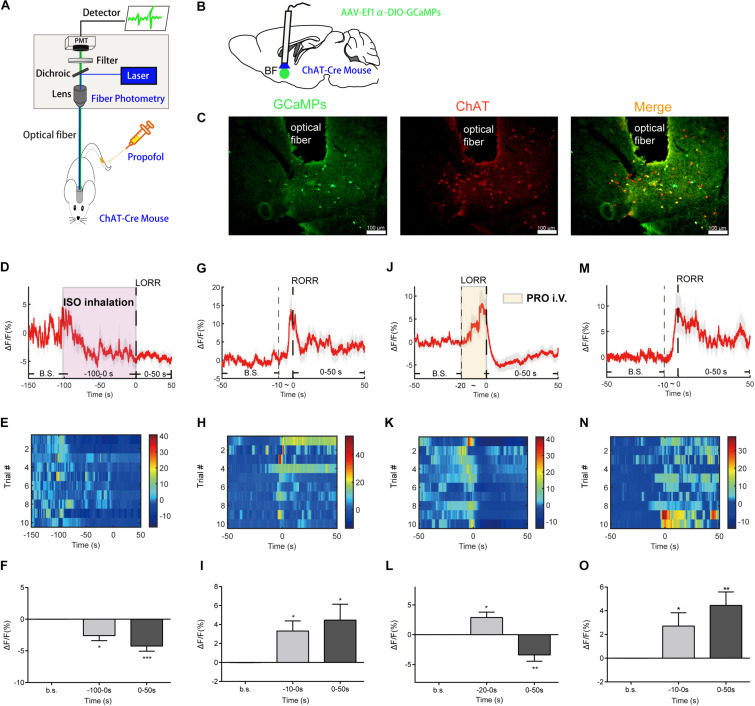
Basal forebrain (BF) cholinergic neurons involved in the procedure of general anesthesia. **(A)** Scheme of fiber photometry setup. **(B)** Scheme of establishing calcium signal recording model in ChAT-Cre mice. **(C)** Expression of GCaMPs (left) in BF ChAT neurons (middle) (scale bar: 100 μm). **(D–F)** Ca^2+^ signals associated with isoflurane-induced loss of righting reflex (LORR). **(D)** Peri-event plot of the average Ca^2+^ transients. The red line indicates the mean, and the shaded (gray) areas indicate SEM. **(E)** Heatmap illustration of Ca^2+^ signals aligned to the initiation of LORR trials. Each row plots one trial, and a total of 10 trials are illustrated. Color scale at the right indicates △F/F. **(F)** Statistical chart of changes in Ca^2+^ signals in isoflurane-induced unconsciousness. △F/F represents the change in GCaMPs fluorescence from the mean level before the isoflurane is given. b.s. is the abbreviation for baseline. **(G–I)** Ca^2+^ signals associated with emergence from isoflurane anesthesia. 0 represents the moment of recovery of righting reflex (RORR). b.s. represents baseline, which is the averaged △F/F between *t* = –50 and –10 s. Time –10 to 0 s shows the changes of Ca^2+^ signals before RORR. **(J–L)** Ca^2+^signals associated with LORR in propofol anesthesia. 0 represents the moment of LORR. b.s. represents baseline, which is the averaged △F/F between *t* = –50 and –20 s. Time –20 to 0 s shows the changes of Ca^2+^ signals during propofol injection. **(M–O)** Ca^2+^ signals associated with the emergence in propofol anesthesia. 0 represents the moment of RORR. b.s. represents baseline, which is the averaged △F/F between *t* = –50 and –10 s. Time –10 to 0 s shows the changes of Ca^2+^ signals before RORR. The *p*-values, compared with baselines, are calculated by using paired *t*-test. *p < 0.05, **p < 0.01, ***p < 0.001 and n.s., not significant.

A multichannel fiber photometry system (Thinker Tech Nanjing Bioscience Inc., China) was used to detect and record the fluorescence signals of GCaMPs. The recording mode is shown in Figures 1A,B. An optical fiber (Newdoon Inc., China) integrated with an optical commutator (Doric Lenses) to guide the light between the fiber photometry system and the implanted optical fiber. The fluorescence signals of GCaMPs were processed separately, filtered at 40 Hz, and digitalized at 500 Hz, and the light intensity at the top of the fiber was set to 20–30 to minimize bleaching. Following a 100-s baseline recording, the mice were administered either a single dosage of propofol (20 mg/kg) *via* the tail vein or 1.4% isoflurane with oxygen at a rate of 1 L/min (Garfield and Bukusoglu, 1996; Cesarovic et al., 2010). Since loss of righting reflex (LORR) and recovery of righting reflex (RORR) are well-established indicators for determining the onset and recovery of general anesthesia in rodent animals (Franks, 2008). The moments of LORR and RORR were marked in the recording process. Ten mice were assigned to the experiment. We performed isoflurane test in all mice prior to the propofol test, and there was at least a 3-day rest between propofol and isoflurane tests. After the experiment, all the mice were sacrificed and the brain tissues were stained with immunofluorescence to detect the viral expression and optical fiber implantation.

### Behavioral and EEG Testing

For propofol anesthesia, we took a single tail vein injection (20 mg/kg) with mouse injection fixation devices (Chuangbo Global Biotechnology Co., Ltd., Beijing) and recorded the duration of anesthesia, that is, the time from LORR to RORR. For isoflurane anesthesia, mice were placed in a recording chamber (RWD Company, Shenzhen, China) filled with 1.4% isoflurane with oxygen at a rate of 1 L/min. The time interval from the start of the isoflurane application to the time when the mice demonstrated LORR for 30 s was determined as the latency to LORR. The mice were kept anesthetized with 1.4% isoflurane for 30 min and then immediately and gently removed from the chamber, and the RORR was quantified in a supine position in room air. The latency to RORR refers to the duration of time when isoflurane stops acting on mice and they resume prostrate and landing on all fours. EEG signals were recorded by a neuronal recording system (Appolo, Bio-Signal Technologies, United States).

In DREADD experiment, Clozapine N-oxide (CNO, Med Chem Express) (1 mg/ml, 3 mg/kg, i.p., based on previous studies; Raper et al., 2017; Snow et al., 2017; Luo Y.J. et al., 2018) or saline (0.9%, equal volume to CNO, i.p.) was injected 1 h before behavior testing and EEG recording (Figures 4A,B). In the optogenetic experiment, an intensity division cube (200 μm/0.37 numerical aperture, Newton Inc., China) was connected to the laser output of a 473 nm optogenetics system (Intelligent Light System, Newdoon Inc., China) and to the mouse-implanted optical fiber for bilateral BF modulation. The optical power at the tip of the fiber was tested with an optical power meter (PM100D, Thorlabs) and calibrated to 10–15 mW. For optical stimulation of BF cholinergic neurons, pulses of 473 nm light with 10 ms width at 20 Hz were applied (Taylor et al., 2016; Zhu et al., 2017). We first tested whether optogenetic activation of BF cholinergic neurons during continuous isoflurane anesthesia can induce behavioral awakening in mice with LORR (Figure 5B). After 1.4% isoflurane induced LORR, the optogenetic activation mode was started. The mice were observed for 5 min to see whether the righting reflex was restored. If there was no change, the isoflurane concentration was reduced by 0.2% every 5 min until it fell to 0.8% concentration, and the behavioral changes of mice were observed throughout the process. We also observed the behavioral changes of optogenetic activation of BF cholinergic neurons for 10 min when the 1.4% isoflurane induced LORR directly adjusted to 0.7% isoflurane for continuous anesthesia (Figure 5C). In addition, we also checked the changes of the latency to LORR and RORR separately. To record the latency to LORR, optical stimulation was initiated simultaneously with 1.4% isoflurane administration, which means that BF cholinergic neurons are activated at the beginning of anesthesia (Figure 5D). To record the latency to RORR, optical stimulation was initiated after anesthesia induction and sustained for 30 min under 1.4% isoflurane (Figure 5E). For propofol anesthesia, optical stimulation began right after propofol induced LORR to determine the LORR duration (Figure 5F).

### Perfusion and Immunofluorescence

For c-fos staining, mice with hM3Dq receptors were injected with CNO (1 mg/ml, 3 mg/kg, i.p.) or saline (0.9%, 3 mg/ml, i.p.) and then kept in their home cage for 2 h before perfusion.

All mice were deeply anesthetized with pentobarbital for the perfusion of 0.01 M phosphate buffered saline (PBS) followed by 4% paraformaldehyde (PFA) after behavioral and EEG testing. The brains were removed and postfixed in PFA overnight at 4°C and then put in PBS with 30% sucrose at 4°C until they sank. The brains were coronally sectioned into 30 μm slices on a cryostat (Leica CM1950).

The brain sections were first incubated in a closed solution (PBS with 2.5% normal goat serum, 1.5% bovine serum albumin, and 0.1% Triton^TM^ X-100) at indoor temperature for 2 h and then coincubated with the primary antibody (c-fos staining, rabbit anti-c-fos, 1:1,000, Synaptic Systems; ChAT staining, AB144P, 1:1,000, Millipore) at 4°C overnight. The sections were then washed with PBST (PBS with 0.1% Triton X-100, vol/vol) on a shaker for several times and incubated with the secondary antibody (goat anti-rabbit Alexa 594 or Alexa 488, 1:1,000, Invitrogen; Donkey anti-goat Alexa 594 or Alexa 488, 1:1,000) for 2 h at indoor temperature. The incubation time reached 2 h, followed by another wash with PBST, and the sections were mounted on glass slides and covered with the installed media [Gold anti-fade reagent with 4’,6-diamidino-2-phenylindole (DAPI), Life Technologies, United States] to be tested. All images were captured by Olympus BX63 virtual microscopy system. The cholinergic neurons and c-Fos-positive cells were counted on alternate sections on both sides of the brain in the BF area (approximately from bregma 0.5 to −0.5 mm, including the horizontal limb of the diagonal band of Broca, magnocellular preoptic nucleus, and substantia innominate, as per the mouse atlas of Paxinos and Franklin, 2001), and the number of cholinergic neurons, mCherry-positive neurons, and c-Fos-positive cells were counted in a 0.8 mm × 0.5 mm box (*n* = 6, 1–2 sections per mouse).

### Data and Statistical Analysis

Using MATLAB 2016a (MathWorks, Cambridge, United Kingdom) to analyze the data measured with fiber-optic sensing. The method of adaptive iteratively reweighted penalized least squares was used to correct baseline drift (Zhang et al., 2010). The change of the fluorescence intensity (ΔF/F) was calculated by using the formula (F–F_0_)/F_0_, where F is the intensity of the measured fluorescence signal, and F_0_ stands for the mean of the fundamental signal. All data were sorted out and recorded as previously described (Li et al., 2016).

EEG signals were digitized and analyzed by using Spike2 software package (Cambridge Electronic Design, Cambridge, United Kingdom). Delta, theta, alpha, beta, gamma, and the total spectral powers were calculated from the 1–4, 5–8, 9–12, 13–25, 26–60, and 1–60 Hz bands, respectively. Then, the averaged signal power across the frequency range of each band was divided by the total power from 1 to 60 Hz to get the relative power.

In this serial study, the data are presented as mean ± SEM. Sample size calculations of every step were based on our previous studies (Luo T. et al., 2018; Zhang et al., 2019). The changes in neuronal calcium signals of different anesthesia stages were analyzed using paired Student’s *t*-tests. The differences in the number of BF cholinergic cells, the latency to LORR or RORR, and the LORR duration between lesioned and control groups were analyzed using unpaired Student’s *t*-test. For the behavior and immunofluorescence staining results of DREADD and optogenetic experiments, we used Student’s paired or unpaired *t*-tests for data analysis when appropriate. For the changes of EEG power bands, two-way ANOVA followed by Bonferroni *post hoc* test was used to analyze data of the lesion and DREADD experiments. In all cases, *p* < 0.05 was considered statistically significant.

## Results

• The activity of BF cholinergic neurons alters during general anesthesia.

To image the neuronal activity of BF cholinergic neurons, we expressed GCaMPs (Resendez and Stuber, 2015), a widely used calcium indicator, in the BF region of ChAT-IRES-Cre mice. GCaMPs was selectively expressed in the BF cholinergic neurons (Figure 1C). During the anesthesia process, *in vivo* Ca^2+^ signals of BF cholinergic neurons were recorded with a calcium fiber photometry system, and the intensity of fluorescence of GCaMPs was used as an indicator.

To study the changes in neuronal calcium signals during the action of isoflurane and propofol, we divide the anesthesia process into three stages according to the transition point of consciousness (LORR or RORR). In detail, for the evaluation of the anesthesia induction process, we divide it into basic stage, induction stage (the time between anesthetics delivery to the moment of LORR), and anesthesia stage (the period after LORR). For the evaluation of anesthesia recovery process, we divide it into basic stage, pre-awakening stage (10 s before RORR), and awakening stage (the period after RORR).

For isoflurane anesthesia, the induction stage is from −100 to 0 s. The Ca^2+^ signal of BF cholinergic neurons declined gradually during the induction stage (*p* = 0.0102, paired *t*-test) and the anesthesia stage (*p* = 0.0006, paired *t*-test), indicating that the cholinergic neuronal activity in BF was suppressed by isoflurane (Figures 1D–F). In the recovery process, the Ca^2+^ signals began to increase during the pre-awakening stage (−10 to 0 s, *p* = 0.0125, paired *t*-test) and peaked approximately at the moment of RORR, then decreased to a certain extent, but remained at a higher level than in the anesthesia state (*p* = 0.0262, paired *t*-test; Figures 1G–I).

For propofol anesthesia, the induction stage is from −20 to 0 s. The activity of BF cholinergic neurons during propofol anesthesia showed a similar trend with isoflurane anesthesia. After a brief increase in propofol injection period (*p* = 0.0107, paired *t*-test, Figures 1J–L), the calcium signal declined rapidly and remained at a low level during the anesthesia stage (*p* = 0.0099, paired *t*-test; Figures 1J–L), indicating that BF cholinergic neurons were inhibited in the anesthesia phase and might be a vital factor for propofol-induced unconsciousness. On the contrary, in the recovery process, the Ca^2+^ signal began to increase in the pre-awakening stage (−10 to 0 s, *p* = 0.0385) and kept at a high level in the awakening stage (*p* = 0.0036, paired *t*-test; Figures 1M–O), indicating that cholinergic neurons in the BF are activated already before the moment of RORR.

The findings show that BF cholinergic neurons participated in the propofol and isoflurane anesthesia. Moreover, since the neuronal signal always altered before behavioral changes, the event of BF cholinergic neurons may be the cause rather than the effect of the change of consciousness state, thus BF neurons might be a target of general anesthetics such as isoflurane and propofol.

• Genetic lesion of BF cholinergic neurons (long-term effects) increased the potency of isoflurane and propofol.

**FIGURE 2:**
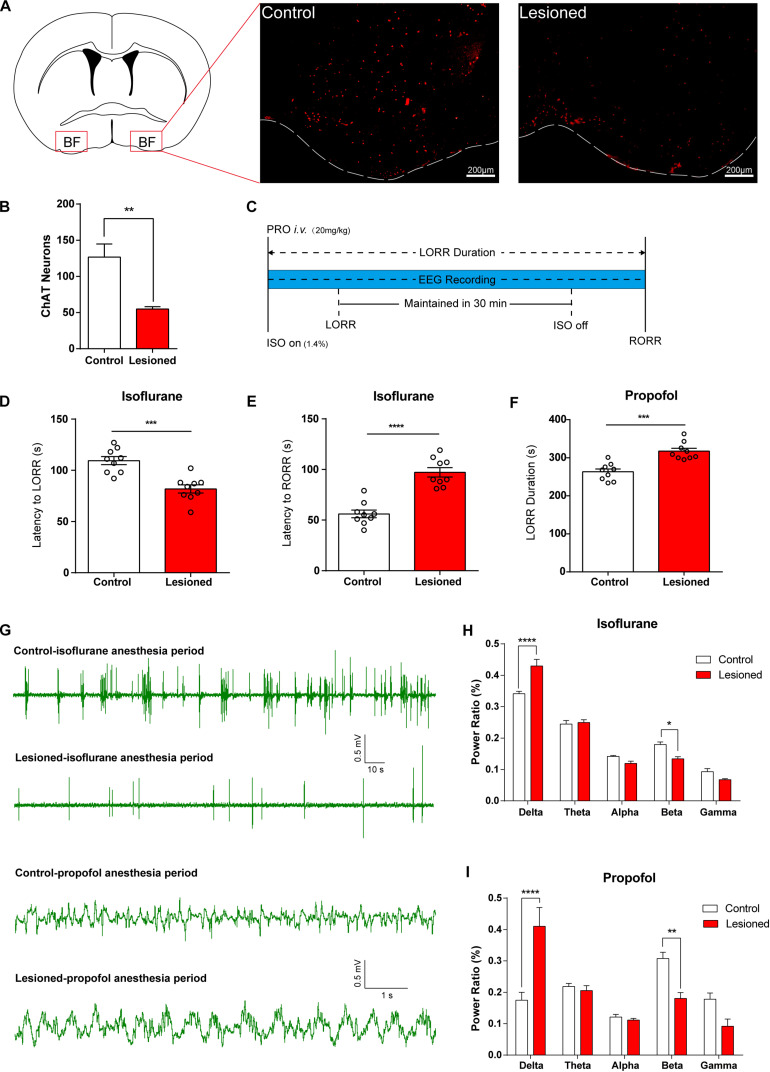
Lesion of basal forebrain (BF) cholinergic neurons enhances general anesthetic efficacy. **(A)** BF cholinergic neurons in saline-injected (left) mice and AAV-CAG-DIO-DTA virus-injected (right) mice (scale bar: 200 μm). **(B)** BF cholinergic cells in the lesioned mice were significantly less than those in the control mice (*p* = 0.0003, *n* = 9, unpaired *t*-test). **(C)** Diagram of behavior testing and EEG recording procedure in propofol and isoflurane anesthesia. **(D,E)** The lesioned group shows significant differences in the latency to loss of righting reflex (LORR) (*p* = 0.0002, *n* = 9, unpaired *t*-test) and the latency to recovery of righting reflex (RORR) (*p* < 0.0001, *n* = 9, unpaired *t*-test) time in isoflurane anesthesia. **(F)** The lesioned group shows a significant increase (*p* = 0.0001, *n* = 9, unpaired *t*-test) in LORR duration time of propofol single dosage injection. **(G)** Representative EEG recording during propofol and isoflurane anesthesia of the lesion and control groups. **(H,I)** Power ratios of EEG recordings from the lesioned and control groups in isoflurane and propofol anesthesia periods, both displaying a significant increase in δ power bands (1–4 Hz) and decrease in β power bands (12–25 Hz). Data are expressed as mean ± SEM, *p < 0.05, **p < 0.01, ***p < 0.001, and ****p < 0.0001.

We further employed three approaches to specifically manipulate BF neural activity to assess its function in general anesthetic efficacy. To verify the necessity of BF cholinergic neurons for regulating anesthetic effects, we induced genetic lesion in the ChAT cells in the BF, which creates a long-term deficit of BF neural activity. We injected AAV-CAG-DIO-DTA virus bilaterally into the BF of ChAT-IRES-Cre mice to selectively express diphtheria toxin (DTA), an extremely strong toxin to cells, in ChAT cells. Figures 2A,B show that the ChAT cells in lesioned mice were significantly less than that in control mice. Two weeks after injection, we assessed the behavioral and electroencephalographic difference between the lesioned mice and the control (saline-injected) mice under either propofol or isoflurane anesthesia (Figure 2C). For isoflurane anesthesia, the BF ChAT neurons lesioned mice took less time to get in the LORR state than control mice (Figure 2D; control group 109.4 ± 3.94 s; lesioned group 81.78 ± 3.99 s; *n* = 9; *p* = 0.0002; unpaired *t*-test), and the latency to RORR in the lesioned group was longer than that of control group (Figure 2E; control group 56.11 ± 3.79 s; lesioned group 97.22 ± 4.64 s; *n* = 9; *p* < 0.0001; unpaired *t*-test). The results of the propofol anesthesia are in accordance with that of the isoflurane anesthesia, the LORR duration [20 mg/kg, intravenously (i.v.)] in the lesioned group is significantly longer than that in the control group (Figure 2F; control group 263.1 ± 7.50 s; lesioned group 317.3 ± 7.63 s; *n* = 9; *p* = 0.0001; unpaired *t*-test). Moreover, deficiency of BF ChAT neurons also impacted cortical EEG in both isoflurane and propofol anesthesia (Figures 2G–I, increase in δ power bands: isoflurane *p <* 0.0001, propofol *p <* 0.0001, and decrease in β power bands: isoflurane *p* = 0.0112, propofol *p* = 0.0041, *n* = 6, Bonferroni’s *post hoc* test).

• DREADD activation of BF cholinergic neurons attenuates the efficacy of general anesthesia.

**FIGURE 3:**
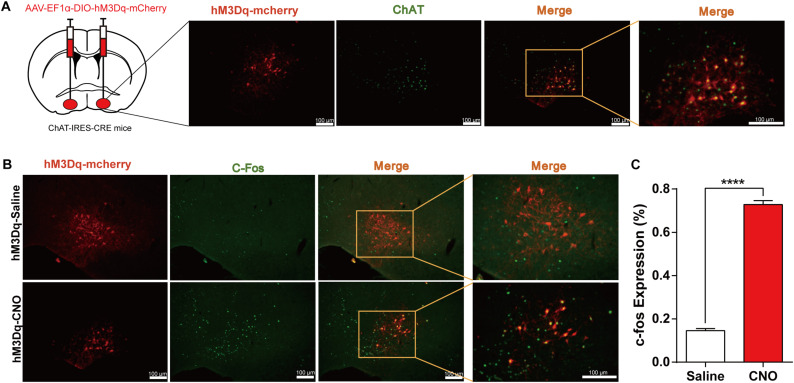
Expression of the hM3Dq receptors in basal forebrain (BF) cholinergic neurons and activation of neurons by Clozapine N-oxide (CNO). **(A)** Bilateral injections of AAV-DIO-hM3Dq-mCherry virus and the expression of hM3Dq-mcherry in BF cholinergic neurons (ChAT positive) (scale bar: 100 μm). **(B)** C-fos expression in cholinergic neurons after hM3Dq mice received CNO or saline intraperitoneal (i.p.) injection (scale bar: 100 μm). **(C)** Quantification of the proportion of mCherry-positive neurons expressing c-Fos under saline and CNO administration [*p* < 0.0001, *n* = 6 (1–2 sections per mouse), unpaired *t*-test]. ****p < 0.0001.

Additionally, to observe how activation of BF cholinergic neurons affects general anesthesia, we employed the DREADD method in which AAV-Ef1α-DIO-hM3Dq-mcherry virus was bilaterally injected into the BF of ChAT-IRES-Cre mice. Immunofluorescence images show that hM3Dq-mcherry is selectively expressed in cholinergic neurons in the BF region (Figure 3A). The expression of c-fos in hM3Dq-mcherry expressing neurons increased significantly after CNO injection [CNO 72.8% ± 1.8% vs. NS 14.5% ± 1.0%, *p <* 0.0001, *n* = 6 (1–2 sections per mouse), unpaired *t*-test; Figures 3B,C], indicating the *in vivo* activation of BF cholinergic neurons by the DREADD method.

**FIGURE 4:**
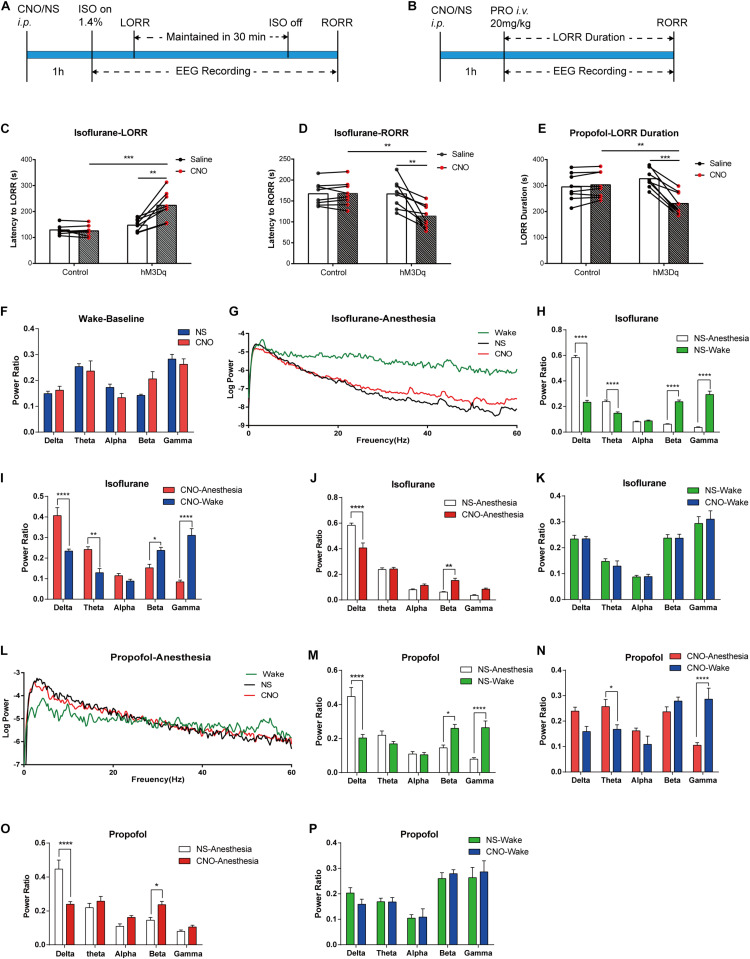
Designer receptor exclusively activated by designer drugs (DREADD) activation of basal forebrain (BF) cholinergic neurons attenuates the efficacy of general anesthesia. **(A,B)** Diagram of DREADD behavior test and EEG recording procedure in isoflurane **(A)** and propofol **(B)** anesthesia. **(C)** The hM3Dq animals injected with Clozapine N-oxide (CNO) show a significant increase in induction time of isoflurane anesthesia (hM3Dq-saline vs. hM3Dq-CNO, *n* = 8, *p* = 0.0028, paired *t*-test; Control-CNO vs. hM3Dq-CNO, *n* = 8, *p* = 0.0003, unpaired *t*-test). **(D)** The hM3Dq animals injected with CNO show a significant decrease in recovery time of isoflurane anesthesia (hM3Dq-saline vs. hM3Dq-CNO, *n* = 8, *p* = 0.0003, paired *t*-test; Control-CNO vs. hM3Dq-CNO, *n* = 8, *p* = 0.0022, unpaired *t*-test). **(E)** Activating BF cholinergic neurons shortened the loss of righting reflex (LORR) duration of propofol anesthesia (hM3Dq-saline vs. hM3Dq-CNO, *n* = 8, *p* = 0.0001, paired *t*-test; Control-CNO vs. hM3Dq-CNO, *n* = 8, *p* = 0.0093, unpaired *t*-test). **(F)** No significant differences were found in baseline EEG between the DREADD animals treated with CNO and control ones. **(G)** Representative EEG recording of log power of isoflurane anesthesia. Wake line (green) is from wake state, NS line (black) is from hM3Dq mice under isoflurane anesthesia injected by saline, CNO (red) line is from hM3Dq mice injected by CNO. **(H,I)** Power ratios of EEG recording in isoflurane anesthesia state and wake state from the hM3Dq-saline group **(H)** and the hM3Dq-CNO group **(I)**. Significant differences between the two states were observed in δ, θ, β, and γ power bands in both groups. **(J,K)** Power ratios of EEG recording from the hM3Dq-saline and hM3Dq-CNO groups in isoflurane anesthesia state **(J)** and wake state **(K)**. A significant decrease in δ power (1–4 Hz, *p* < 0.0001, *n* = 6, Bonferroni’s *post hoc* test) and increase in β power (12–25 Hz, *p* = 0.0012, *n* = 6, Bonferroni’s *post hoc* test) were displayed in the anesthesia state. No significant differences were found in the wake state between the two groups. **(L)** Representative EEG recording of log power of propofol anesthesia. Wake line (green) is from wake state, NS line (black) is from hM3Dq mice under propofol anesthesia injected by saline, CNO (red) line is from hM3Dq mice injected by CNO. **(M,N)** Power ratios of EEG recording in propofol anesthesia state and wake state from the hM3Dq-saline group **(M)** and the hM3Dq-CNO group **(N)**. Significant differences between the two states were observed in δ, β, and γ power bands in the hM3Dq-saline group and θ and γ bands in the hM3Dq-CNO group. **(O,P)** Power ratios of EEG recording from the hM3Dq-saline and hM3Dq-CNO groups in propofol anesthesia state **(O)** and wake state **(P)**. Differences of δ power (1–4 Hz, *p* < 0.0001, *n* = 6, Bonferroni’s *post hoc* test) and β power (12–25 Hz, *p* = 0.0443, *n* = 6, Bonferroni’s *post hoc* test) are found between the hM3Dq-saline and hM3Dq-CNO groups in the anesthesia state. No significant differences were found in the wake state between the two groups. Data are expressed as mean ± SEM, *p < 0.05, **p < 0.01, ***p < 0.001, and ****p < 0.0001.

For isoflurane anesthesia, the DREADD group required a longer induction time to fall in anesthesia state (Figure 4C; hM3Dq-CNO group 224.0 ± 19.32 s, hM3Dq-saline group 147.38 ± 8.60 s, *n* = 8, *p* = 0.0028, paired t-test; control-CNO group 125.5 ± 7.18 s, *n* = 8, *p* = 0.0003, unpaired *t*-test), and the recovery time was shorter (Figure 4D; hM3Dq-CNO group 113.63 ± 9.95 s, hM3Dq-saline group 116.75 ± 12.14 s, *n* = 8, *p* = 0.0003, paired *t*-test; control-CNO group 125.5 ± 7.18 s, *n* = 8, *p* = 0.0022, unpaired *t*-test). To eliminate the effect of CNO or virus on general anesthesia, we also set AAV-Ef1α-DIO-mCherry-expressed mice as the control group, and no behavioral difference was found between the control groups treated with CNO and saline. No significant differences were found in baseline EEG between the DREADD group and the control group (*p* > 0.05, *n* = 6, Bonferroni’s *post hoc* test; Figure 4F), and there was also no significant EEG difference between the two groups after recovery from isoflurane (Figure 4K) or propofol (Figure 4P) anesthesia. Isoflurane anesthesia exerted a profound influence on the EEG spectrum of the control group (Figure 4H) and the DREADD group (Figure 4I), including δ, θ, β, and γ bands (*n* = 6, Bonferroni’s *post hoc* test). However, the DREADD group shows lower δ (*p* < 0.0001, *n* = 6, Bonferroni’s *post hoc* test) and higher β (*p* = 0.0012, *n* = 6, Bonferroni’s *post hoc* test) power bands than those of the control group (Figures 4G,J).

For propofol anesthesia, the LORR duration is obviously shortened in hM3Dq-CNO mice compared with that in the hM3Dq-saline group and control group (Figure 4E; hM3Dq-CNO group, 230.8 ± 15.93 s; hM3Dq-saline group, 326.3 ± 12.79 s, *n* = 8, *p* = 0.0001, paired *t*-test; control-CNO group, 302.8 ± 17.81 s, *n* = 8, *p* = 0.0093, unpaired *t*-test). There was no significant difference between the control groups treated with CNO or saline (control-CNO group, 302.8 ± 17.81 s; control-saline group, 294.6 ± 18.79 s, *n* = 8, *p* = 0.098, paired *t*-test). Propofol anesthesia also changed the EEG spectrum of the control group and the DREADD group. In detail, the control-saline group showed an increase in the δ frequency band and a decrease in the θ and γ frequency bands (Figure 4M), and the hM3Dq-CNO group showed a decrease in the γ band and an increase in θ band (Figure 4N). In comparison, in the hM3Dq-CNO group, the EEG δ power bands (1–4 Hz) significantly decreased (*p* < 0.0001, *n* = 6, Bonferroni’s *post hoc* test; Figures 4L,O) and the β power bands (12–25 Hz) increased (*p* = 0.0443, *n* = 6, Bonferroni’s *post hoc* test; Figures 4L,O) when compared with those in the hM3Dq-saline group, suggesting that the depth of propofol anesthesia was attenuated by activating BF cholinergic neurons.

• Optogenetics activation of BF cholinergic neurons (instantaneous manipulation) on general anesthesia induction and emergence.

**FIGURE 5:**
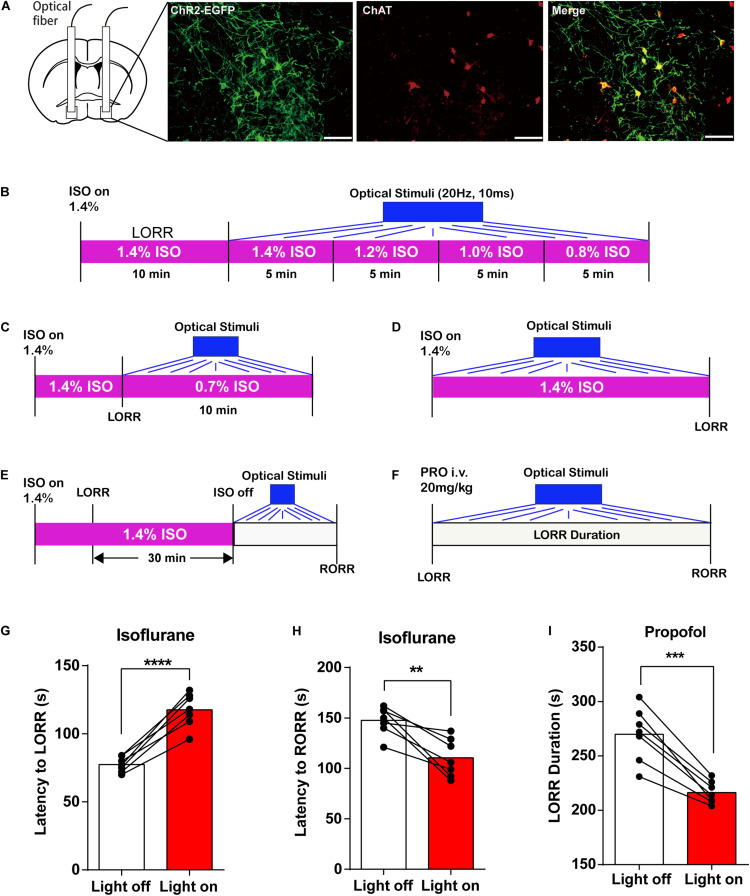
Optogenetic activation of basal forebrain (BF) cholinergic neurons on general anesthesia induction and emergence. **(A)** EGFP (green) expressed in BF cholinergic neurons (red) after bilateral injections of AAV-DIO-ChR2-EGFP virus (scale bar: 50 μm). **(B)** Diagram of optogenetic behavior test under isoflurane anesthesia with an isoflurane concentration gradient from 1.4 to 0.8%. **(C)** Diagram of optogenetic behavior test under 0.7% isoflurane anesthesia. **(D)** Diagram of optogenetic behavior test procedure of propofol anesthesia. **(E)** Diagram of optogenetic behavior test procedure during the induction of isoflurane anesthesia. **(F)** Diagram of optogenetic behavior test procedure during the emergence of isoflurane anesthesia. **(G,H)** Optogenetic activation of BF cholinergic neurons increased the latency to loss of righting reflex (LORR) time (*p* < 0.0001, *n* = 7, paired *t*-test) and decreased the latency to recovery of righting reflex (RORR) (*p* = 0.0034, *n* = 7, paired *t*-test) in isoflurane anesthesia. **(I)** Optogenetic activation of BF cholinergic neurons decreased propofol LORR duration time (*p* = 0.0002, *n* = 7, paired *t*-test). Data are expressed as mean ± SEM, **p < 0.01, ***p < 0.001, and ****p < 0.0001.

The above experiments showed that BF cholinergic neurons participated in the regulation of general anesthetic efficacy. We further asked whether transient manipulation of BF activity can directly reverse the anesthetic effect. Optogenetics activation method was applied to transiently active BF cholinergic neurons. We infused the virus (AAV-Ef1α-DIO-hChR2-EYFP) and implanted optic fibers bilaterally into the BF in ChAT-IRES-Cre mice. The virus expression and the co-labeling with ChAT are shown in Figure 5A.

We first applied optical stimuli to activate BF cholinergic neurons under constant anesthesia with a concentration gradient from 1.4 to 0.8% (Figure 5B) but failed to induce RORR. Thus, we lowered the isoflurane concentration to 0.7% (Figure 5C) and still failed to induce RORR. However, the induction and emergence time under the optical stimuli exhibit similar trends with those in the DREADD experiment (Figures 5G–I; isoflurane: the latency to LORR, lights off 79.57 ± 2.256 s, lights on 116.1 ± 4.522 s, *n* = 7, *p* < 0.0001; the latency to RORR, lights off 147.6 ± 5.304 s, lights on 110.4 ± 7.201 s, *n* = 7, *p* = 0.0034; propofol: LORR duration, lights off 269.9 ± 9.39 s, lights on 216.3 ± 3.908 s, *n* = 7, *p* = 0.0002; paired *t*-test). The findings indicated that BF cholinergic neurons regulate the propofol and isoflurane anesthesia but may not be the only determinant of the anesthesia process.

## Discussion

With the help of various approaches based on transgenic mice including calcium fiber photometry, genetic lesion, DREADD, optogenetics activation, and neural electrophysiology, we found that both isoflurane and propofol inhibit the event of BF cholinergic neurons, which are associated with consciousness states. The cell-specific lesions of mouse BF cholinergic neurons were responsible for a markedly increased potency of anesthetics, shown as a quicker induction time or a longer recovery time and a significant increase in EEG delta power. Accordingly, activated BF cholinergic neurons by DREADD reduced the anesthetics’ sensitivity with a longer time to LORR for isoflurane, a faster RORR for both drugs, and reduced EEG delta power. Moreover, optical stimulation of BF cholinergic cells also reduced anesthetic sensitivity but failed to restore wake-like behavior in constantly anesthetized mice. The results indicate that BF cholinergic neurons make for isoflurane and propofol anesthesia in both behavioral and cortical levels.

To determine whether a nucleus is involved in general anesthesia, lesion method used to be the most common and only approach (Leung et al., 2011, 2013; Tai et al., 2014). However, the effects of general anesthetics on neuronal activity are often overlooked due to the inherent limitation of the technique. Technical improvement allows us not only to clarify the responses of neuronal activity to anesthetics *in vivo* but also to identify the functional role of such responses. We employed calcium fiber photometry system, which is one of the most sensitive methods for recording neuronal activities in the deep part of the brain (Cox et al., 2016; Pisanello et al., 2019), to record anesthesia-related BF cholinergic neuronal activities. The results that BF cholinergic neurons are activated during arousal and inhibited during anesthesia are in accordance with what has been shown in the sleep–wake process (Greco et al., 2000). Moreover, the neuronal activity alters before behavioral changes in the emergences from isoflurane and propofol anesthesia, indicating that cholinergic neurons in the BF might be the initial point or some key part in the pathway to induce the recovery from general anesthesia. We also discovered that continuous isoflurane gradually inhibits the activity of cholinergic neurons in the induction period, and LORR occurred when the activity reduced to a certain level. However, for propofol anesthesia, we noticed that the injection of propofol causes a brief and slight activation of BF cholinergic neurons; this is likely on account of the direct stimulation of injection pain or signal transmission from other pain sensation-related brain regions (Yuan et al., 2016; Luo T. et al., 2018). In spite of this, the event of BF cholinergic neurons is rapidly and continuously inhibited during propofol anesthesia. A study on brain slices also confirmed that propofol could decrease the excitability of cholinergic neurons in the basal forebrain of mouse (Chen et al., 2019). These results suggest that the BF cholinergic system might serve as a critical target for the function of general anesthetics.

Subsequently, we specifically destroyed BF cholinergic neurons by combining transgenic mice with a specific destructive virus. Compared to the traditional chemical method, this approach has the merits of cell specificity and accuracy. BF cholinergic neuron lesioned mice were more sensitive to propofol and isoflurane in both behavior and EEG performance, and they have a longer recovery time, which are consistent with Leung’s findings with rats (Leung et al., 2011; Tai et al., 2014). However, there are still concerns about the lesion approach, as it has reported that lesion experiment of the sleep–wake nucleus may lead to changes in sleep debt, suggesting that the lesion itself might affect anesthetic efficacy (Tung et al., 2002). The ventrolateral preoptic nucleus (VLPO), a natural sleep-promoting center, has been reported to have an anesthetic enhancement rather than attenuating effect with a lesion experiment of 24 days (Moore et al., 2012). Therefore, to confirm the accurate functions of neurons, we employed DREADD and optogenetic approaches, which manipulate specific neuronal activity for shorter term, thus eliminating the harm to the nucleus.

The DREADD results show that activated BF cholinergic neurons could resist general anesthesia, manifested by prolonged LORR induction time of isoflurane and shortened LORR duration of propofol. An acceleration of recovery from isoflurane anesthesia was also observed with BF cholinergic activation. The EEG recordings of DREADD-stimulated BF cholinergic neurons under propofol and isoflurane anesthesia illustrated a significantly decreased δ power and an increased β power. No significant differences were observed in other power bands. This is in accordance with previous studies that activated BF cholinergic neurons destabilize the sleep state and leads to fragmentation in endogenous sleep and also facilitates the waking EEG by suppressing the lower frequency components (Irmak and de Lecea, 2014; Anaclet et al., 2015). Our results further support that the sleep–wake cycle and anesthesia shared some of the same mechanisms through BF cholinergic neurons. The results also suggest that BF cholinergic neurons themselves participated in the regulation of anesthetic effect rather than the concerned sleep debt. The corresponding changes of EEG δ power after activation or lesion of BF cholinergic neurons may be caused by the release of acetylcholine in the cortex from terminals of these neurons. According to a report, the slow decrease of acetylcholine in the cortex is positively correlated with the excitability of cortical pyramidal neurons and rhythmic burst discharge, facilitating higher-frequency, single-spike discharges in burst-generating neurons (Metherate et al., 1992). Moreover, cholinergic stimulation of the prefrontal cortex that receives ascending cholinergic projection from the BF was reported to induce RORR behavior in anesthetized rats (Pal et al., 2018). These studies not only provide more evidence on the functional role of the cholinergic system in regulating anesthetic effect but also suggest that BF cholinergic neurons may exert such effects through the downstream prefrontal cortex.

The results of optogenetic activation were similar to those of DREADD. However, acute activation of BF cholinergic neurons by optogenetic method cannot directly induce reanimation from continuous isoflurane administration, even at a concentration as low as 0.7%. The findings are consistent with those of a previous study that found that physostigmine, a centrally acting cholinesterase inhibitor, failed to restore consciousness in rats exposed to 0.9% isoflurane (Kenny et al., 2016). Our study further supports that cholinergic activation in not all brain regions can induce reanimation from anesthetized animals (Pal et al., 2018), which also helps explain why physostigmine in the previous study was unable to restore righting during continuous isoflurane administration (Kenny et al., 2016). To date, activation of several brain regions has been reported to directly induce RORR in animals undergoing constant anesthesia. Electrical stimulation of the parabrachial nucleus (PBN) and optogenetic activation of dopamine neurons in the ventral tegmental area (VTA) both induce reanimation from isoflurane anesthesia (Muindi et al., 2016; Taylor et al., 2016); in addition, stimulation of the prefrontal cholinergic system by reverse dialysis delivery of 5 mM carbachol induces wake-like behavior in constant sevoflurane-anesthetized rats (Pal et al., 2018). Unlike these neurons, which play a decisive role in general anesthesia, cholinergic neurons of the BF may contribute to general anesthesia only through regulatory functions. However, there are good anatomical and functional connections between BF and aforementioned critical areas. It has been reported that BF is a crucial downstream pathway for PBN to exert arousal regulation, though there are other pathways (Qiu et al., 2016). BF cholinergic neurons have also been shown to receive dopaminergic projections from the VTA region that regulates wakefulness (Gaykema and Zaborszky, 1996). These studies indicate that the BF is the intermediate point of contact for many of the brain stem nuclei that control arousal. In addition, the BF projects widely down to the cortex (Bloem et al., 2014), thus the awakening effect of the cholinergic nervous system activation in the prefrontal cortex (Pal et al., 2018) may anatomically derive from the BF cholinergic neurons.

Although there are some differences between propofol and isoflurane in terms of administration methods and molecular targets (Franks, 2008), our study found that BF cholinergic neurons regulate the anesthetic effects of the two drugs in the same way, which may indicate that both isoflurane and propofol ultimately act on the arousal neural network where BF cholinergic neurons are located through their respective molecular targets. In addition, there are some brain areas that have consistent regulatory effects on the efficacy of different anesthetics, such as locus coeruleus, medial septum, and hippocampus (Leung et al., 2014). These results suggest that the neuronal network may be the key to decipher the mechanism of action of general anesthetics.

Despite the regulatory role in general anesthesia, BF is reported to be one of the few nuclei in the brain that induce animals into a coma state by direct damage (Fuller et al., 2011), suggesting that the BF is an indispensable wake-promoting nucleus. Moreover, recent studies have found that the BF controls the sleep–wake state through four types of neurons, including cholinergic neurons (Xu et al., 2015). In addition, studies have reported that BF cholinergic neurons facilitate wakefulness by acting on adjacent non-cholinergic neurons (Zant et al., 2016). This helps explain the regulatory rather than the conclusive action of BF cholinergic neurons in anesthesia. The functions of BF non-cholinergic nerves in general anesthesia are to be investigated.

In clinical practice, the elderly and Alzheimer’s disease patients usually have high anesthesia sensitivity and are prone to delayed recovery (Funder et al., 2009). Our study may partially explain this phenomenon because these patients are usually accompanied by a significant decrease in BF cholinergic neurons (McGeer et al., 1984).

## Conclusion

In conclusion, this report underscores that BF cholinergic neurons are vital for promoting arousal in both isoflurane and propofol general anesthesia, and we deduced that BF cholinergic neurons are involved in the pathway that is crucial to regulate the efficacy of general anesthetics. In order to deepen our understanding of BF-related mechanisms of general anesthesia, it is necessary to further study the function of BF non-cholinergic neurons in mediating the anesthesia state.

## Data Availability Statement

Datasets generated for this study are available from the corresponding author on reasonable request.

## Ethics Statement

The animal study was reviewed and approved by the animal protection and utilization committees of Zunyi Medical University, Guizhou, China.

## Author Contributions

SC, Z-XQ, S-CY, YS, and C-XL: methodology. T-YL, LZho, and S-YY: formal analysis. LZha and YZ: investigation. T-YL and SC: writing—original draft. S-YY and TY: writing—review and editing. All authors read and approved the final manuscript.

## Conflict of Interest

The authors declare that the research was conducted in the absence of any commercial or financial relationships that could be construed as a potential conflict of interest.
